# Frequency of periodontal pathogens and *Helicobacter pylori* in the mouths and stomachs of obese individuals submitted to bariatric surgery: a cross-sectional study

**DOI:** 10.1590/1678-775720150534

**Published:** 2016

**Authors:** André Luiz PATARO, Sheila Cavalca CORTELLI, Mauro Henrique Nogueira Guimarães ABREU, José Roberto CORTELLI, Gilson Cesar Nobre FRANCO, Davi Romeiro AQUINO, Luis Otavio Miranda COTA, Fernando Oliveira COSTA

**Affiliations:** 1 - Universidade Federal de Minas Gerais, Departamento de Periodontia, Belo Horizonte, Minas Gerais, Brasil.; 2 - Universidade de Taubaté, Núcleo de Pesquisa Periodontal, Departamento de Odontologia, Taubaté, São Paulo, Brasil.; 3 - Universidade Estadual de Ponta Grossa, Laboratório de Fisiologia e Patofisiologia, Departamento de Biologia Geral, Ponta Grossa, Paraná, Brasil.; 4 - Universidade Federal de Minas Gerais, Departamento de Odontologia Social e Preventiva, Belo Horizonte, Minas Gerais, Brasil.

**Keywords:** Obesity, Bariatric surgery, Periodontal diseases, Bacteria

## Abstract

**Objectives:**

This cross-sectional study compared the frequency of oral periodontopathogens and *H. pylori* in the mouths and stomachs of obese individuals with or without periodontitis submitted to bariatric surgery.

**Material and Methods:**

One hundred and fifty-four men and women aged 18-65 were conveniently distributed into four groups. Two groups were composed of individuals who underwent bariatric surgery with (BP) (n=40) and without (BNP) (n=39) periodontitis and two obese control groups with (CP) (n=35) and without (CNP) (n=40) periodontitis. The oral pathogens *Porphyromonas gingivalis*, *Aggregatibacter actinomycetemcomitans*, *Parvimonas micra*, *Treponema denticola*, *Tannerella forsythia*, *Campylobacter rectus*, and *Helicobacter pylori* were detected by a polymerase chain reaction technique using saliva, tongue and stomach biopsy samples.

**Results:**

Statistical analysis demonstrated that periodontopathogens were highly frequent in the mouth (up to 91.4%). In the bariatric surgically treated group, orally, *P. gingivalis*, *T. denticola* and *T. forsythia* were more frequent in periodontitis, while *C. rectus* was more frequent in non-periodontitis subjects. Stomach biopsies also revealed the high frequency of five oral species in both candidates for bariatric surgery (91.6%) and the bariatric (83.3%) groups. *H. pylori* was frequently detected in the mouth (50.0%) and stomach (83.3%). In the stomach, oral species and *H. pylori* appeared in lower frequency in the bariatric group.

**Conclusions:**

Obese individuals showed high frequencies of periodontopathogens and *H. pylori* in their mouths and stomachs. Bariatric surgery showed an inverse microbial effect on oral and stomach environments by revealing higher oral and lower stomach bacterial frequencies.

## INTRODUCTION

High prevalence of obesity and its complications have become a global health concern. Besides different systemic comorbidities^16^, obesity has been linked to oral status and specifically to an increased risk for the development and severity of periodontal disease^7^ ^,^ ^8^ ^,^ ^20^ ^,^ ^22^ ^,^ ^23^ among the most prevalent oral diseases in different populations. However, a recent systematic review pointed out that evidence on this subject is still limited^21^. Periodontitis triggers local and systemic variations in pathophysiology mechanisms that lead to a chronic inflammatory state, thereby increasing susceptibility to metabolic syndromes. In addition, given that the adipose tissue, particularly the white adipose tissue, acts as a main endocrine organ secreting a number of bioactive substances (such as cytokines), it can also affect the periodontal response and it can also be affected by periodontal infections^20^.

Periodontal disease is a multifactorial infectious disease associated with a microbiota predominantly composed of Gram-negative species. The link between obesity and periodontal disease is based on inflammatory characteristics. Although starting agents could be different, both show high levels of inflammatory mediators^18^ ^,^ ^20^. Despite its relevance, the number of surveys that have attempted to analyze the relationship between obesity and periodontal microbiota is still small. However, a few preliminary studies have found different oral microbial profiles after comparing obese and non-obese groups^10^ ^,^ ^11^, therefore indicating the need for further studies in this area. Although Belstrøm, et al.^3^ (2014) failed to establish an association between body mass index and salivary bacterial profiles, the Human Microbiome project has shown that shifts in our microbiota are associated with many diseases such as obesity^14^. Recently, a cross-sectional study performed in Japan revealed an association between *Porphyromonas gingivalis*, *Treponema denticola,* and *Tannellera forsythia* and obesity^17^.

Although microbial studies focusing on obesity and periodontal status are limited, when obesity and bacterial frequency are mentioned, *Helicobacter pylori* undoubtedly plays a key role. Oral sites could represent a reservoir for *H. pylori* ^9^ ^,^ ^28^ and, in theory, the mouth-stomach route used by this pathogen can also be used by periodontal pathogens to access other parts of the body. Bariatric surgery is a commonly recommended treatment option for severely obese subjects^19^. After surgery, several personal changes can be observed; however, the oral microbial profile of bariatric surgery patients remains controversial^24^ ^,^ ^30^. Although there have been reports on the possible association between obesity and periodontal disease, specific data on the influence of obesity and/or bariatric surgery on stomach frequency of periodontal pathogens had not been gathered yet. Moreover, there are no reports in the literature describing whether periodontal pathogens can translocate from the mouth to colonize the gastric mucosa. Therefore, the present study was performed with the aim of evaluating the oral, and especially stomach, presence of certain target periodontal pathogens and *H. pylori* in bariatric surgically treated obese individuals.

## MATERIAL AND METHODS

### Participants

One hundred and fifty-four obese adult individuals (121 females and 33 males, 37.58±11.36 years of age) recruited from two health centers for the treatment of obesity (Trauma One Clinic located in Belo Horizonte, Minas Gerais, Brazil and Dental Medic Clinic located in Lorena, São Paulo, Brazil) composed the population of this study. All of them underwent a complete periodontal clinical examination between December 2009 and December 2013. Inclusion criteria were adults between 18 and 65 years of age, both genders, who had been bariatric surgically treated by the gastric bypass Roux-en-Y – Fobi-Capella technique, and obesity (BMI>30 and <40) for the control groups. In addition, the diagnosis of periodontitis (described *a posteriori*) was a requirement for two groups while a non-periodontitis diagnosis was an inclusion criterion for the other two groups. The exclusion criteria were: those aged under 18 or over 65; underweight Body Mass Index (BMI<19 for surgically treated patients) or morbid obesity (BMI>40); possession of fewer than 15 natural teeth; pregnancy; antibiotic intake or regular use of chlorhexidine within the three months previous to the examination.

Participants were carefully informed about the objectives of the study and after their approval they signed a consent form. This study was approved by the Federal University of Minas Gerais Research Committee (ETIC 57807) and University of Taubaté Ethics Research Committee (protocol 52210).

The study population was conveniently distributed in four distinct groups. The first included the bariatric surgically treated groups composed of patients that undergone the surgery at least 24 months later (39.37±15.80 months after surgery). This group was subdivided according to the presence of periodontitis (bariatric with periodontitis - BP) or absence of periodontitis (bariatric with no periodontitis - BNP) and was composed of 40 and 39 individuals, respectively. The second included two obese groups: one composed of obese patients showing signs of periodontitis (control with periodontitis - CP) and the other composed of obese patients without signs of periodontitis (control with no periodontitis - CNP). These groups were composed of 35 and 40 individuals, respectively. The number of 32 individuals per group, as the minimum needed, was determined based on results from a pilot study, which included sampling and laboratorial processing of 10 obese individuals. Statistics revealed that this number would be enough to identify a minimum significant difference of 10%. In addition, a 10% safety margin was adopted.

Data regarding demographic information and oral hygiene habits were collected from each participant while their medical histories were obtained from their medical records. Anthropometric measurements, including weight (Kg) and height (m), were measured while the subjects were wearing light clothing and no shoes by a center trained and calibrated professional nutritionist. These measurements were used to calculate BMI (Kg/m^2^) and classify obesity^29^. Twenty individuals had their height and weight re-measured to determine BMI reproducibility values. The Kappa test showed values greater than 0.90, thus indicating good reproducibility.

### Periodontal clinical examination

For each participant, a full-mouth periodontal examination was performed in a hospital gurney with a photophore. Two periodontists (A.L.P. and S.C.C), trained and calibrated at the beginning of the study, measured probing depth (PD) and clinical attachment level (CAL). After seven days, the periodontal examinations of 10 participants were repeated, showing intra- and inter-examiner reproducibility scores that were higher than 0.85 (Kappa test) for PD and CAL clinical parameters. Intra-class correlation tests showed scores higher than 0.90. The following periodontal parameters were registered with a manual periodontal probe (North Carolina University model – UNC – #15, Hu-Friedy; Chicago, IL, USA) at six sites per tooth: bleeding on probing (BOP), PD and CAL. Periodontitis was defined as ≥4 teeth with ≥1 site showing, simultaneously, a probing depth ≥4 mm and clinical attachment level ≥3 mm^15^. Oral hygiene was assessed using plaque index (PI)^26^.

### Microbiological assessment

Samples of saliva^6^ and from the dorsum of the tongue^5^ were collected from the total population (n=154). Saliva samples were collected in the morning, between 8:00 and 11:00. The patients were instructed not to eat or drink prior to sampling. Immediately before sampling, individuals rinsed their mouths with water. During collection, they remained seated with their heads tilted forward (approximately 45°) and 2.0 mL of unstimulated, whole saliva were collected into sterile Falcon tubes. Samples were centrifuged for 10 minutes at 15,000×g at 4°C, and the supernatants were immediately stored at -80°C. Scrapings from the tongue dorsum were taken from areas of approximately 1 cm^2^ using a cotton swab dipped in reduced Ringer’s solution, rotated six times. Each swab was transferred into a microtube also containing reduced Ringer’s solution (1 mL).

According to systemic medical needs, additional stomach biopsies were collected from a representative subgroup of 49 subjects requiring endoscopy. Following the protocols of the medical centers, a gastroenterologist physician collected the stomach biopsies using an endoscope. Tissue samples were inserted into Eppendorf microtubes containing 1 mL of reduced Ringer’s solution and were immediately placed in a polystyrene box with ice and then stored at low temperatures (-20°C) until processing.

Microbial processing procedures were performed as previously described^5^. Each collected sample was immediately conditioned in a Styrofoam box with ice and then stored at low temperatures (-80°C) until processing. The genomic DNA of each sample was extracted using PureLink™ Genomic DNA Purification Kit (Invitrogen, Carlsbad, CA, USA) according to the manufacturer’s instructions. Prior to specific microbial analysis, a polymerase chain reaction (PCR) was carried out using unspecific “Universal primers” (16S rRNA) (5’-GATTAGATACCCTGGTAGTCCAC-3’ and 5’-CCCGGGAACGTATTCACCG-3’) to detect bacterial DNA in the samples. After this procedure, the presence of *P. gingivalis* (5’-AGGCAGCTTGCCATACTGCG-3’ and 5’-ACTGTTAGCAACTACCGATGT-3’), *Aggregatibacter actinomycetemcomitans* (5’-ATGCCAACTTGACGTTAAAT-3’ and 5’-AAACCCATCTCTGAGTTCTTCTTC-3’), *Parvimonas micra* (5’- GTAATGATGGGGACTCTGGA-3’ and 5’- CTTCCTCCTTGCGGTTAGAT -3’), *T. denticola* (5’- TAATACCGAATGTGCTCATTTACAT-3’ and 5’- TCAAAGAAGCATTCCCTCTTCTTCTTA -3’), *T. forsythia* (5’-GCGTATGTAACCTGCCCGCA-3’ and 5’-TGCTTCAGTGTCAGTTATACCT-3’), *Campylobacter rectus* (5’-TTTCGGAGCGTAAACTCCTTTTC-3’ and 5’-TTTCTGCAAGCAGACACTCTT-3’), and *H. pylori* (5’- GAGCGCGTAGGCGGGATAGTC-3’ and 5’- CGTTAGCTGCATTACTGGAGA -3’) was established using specific primers under standard conditions. PCR was performed using a Mastercycler Gradient (Eppendorf^®^, Westbury, NY, USA) thermocycler as follows: one cycle at 94°C for 5 minutes; 35 cycles at 94°C for 30 seconds, 55-60°C for 30 seconds, and 72°C for 1 minute; and a final cycle of 72°C for 5 minutes. After electrophoresis in 1.5% agarose gel, the DNA fragments were stained with SYBR Safe (Invitrogen, Carlsbad, CA, USA) and visualized by UV illumination. The PCR amplifications were compared with both positive and negative controls. A molecular weight marker (Ladder 100, Invitrogen, Carlsbad, CA, USA) was added in each set. To ensure PCR reproducibility, 20% of the samples were re-amplified.

### Statistical analysis

The frequency of each bacterium was separately evaluated in the samples taken from the saliva and dorsum of the tongue. The occurrence of each bacterium in a given subject in at least one of these sites was also checked. This last evaluation was referred to as oral representation. An additional analysis considering the red complex oral bacterial species was also performed, thereby indicating the simultaneous presence of *P. gingivalis, T. denticola* and *T. forsythia*.

The comparison of the frequencies of bacteria verified in this study was made using the Pearson Chi-square and Fisher’s test. To determine the significance between groups, the Bonferroni correction was applied. The characteristics of the participants’ variables were described using frequency distribution for categorical variables and median, mean and standard deviation for continuous variables. Normality data distribution was verified by the Kolmogorov-Smirnov test. The ANOVA test was used for variables of normal distribution and the Kruskal-Wallis test was used for those of non-normal distribution, followed by the Tukey’s test and the Mann-Whitney test for comparisons between groups. Tests of accuracy tested the relationships between the stomach and oral frequencies of each target bacterial species.

All statistical tests were performed using SSPS (Statistical Package for Social Sciences, version 16 for Windows) software (SPSS Inc., Chicago, IL, USA) and considered significant for p-values <0.05.

## RESULTS

As expected, BMI was greater among the controls when compared with the bariatric groups. In addition, within the periodontal groups (with or without periodontitis), BMI values were similar (Table 1).

**Table 1:** Body Mass Index (BMI) and periodontal clinical variables from control groups or bariatric groups according to periodontal status (mean±standard deviation and median)

	CP	CNP	BP	BNP	TOTAL	p-value
	n=35	n=40	n=40	n=39	n=154	
Body mass index	41.65±4.70^a^ 40.4	39.89±7.08^a^ 39.6	26.89±4.48^b^ 26.4	26.53±4.23^b^ 25.2	33.53±8.77 32.4	<0.001^γ^
Bleeding on probing	28.64±12.08 29	20.33±19.43 14.9	30.26±20.37 23	24.67±22.57 18.7	25.90±19.39 21.75	0.102^γ^
Plaque index	1.09±0.34	1.04±0.72	1.04±0.48	1.02±0.43	1.05±0.51	0.212*
	1.00	0.83	1.00	1.00	1.00	
Sites ≥PD 4 mm	10.75±5.70^c^ 9.61	1.33±1.27^d^ 0.92	12.72±9.95^c^ 9.72	0.68±0.85^d^ 0.00	6.26±7.93 3.2	<0.001*
Sites ≥PD 5 mm	3.44±3.34^c^ 2.67	0.19±0.56^d^ 0.00	4.75±5.52^c^ 1.85	0.12±0.39^d^ 0.00	2.10±3.81 0.00	<0.001
Sites ≥CAL 4 mm	15.53±10.45^c^ 13.39	3.72±6.73^d^ 1.82	16.31±14.56^c^ 11.25	3.52±5.57^d^ 1.78	9.62±11.66 5.35	<0.001 *
Sites ≥CAL 5 mm	6.60±7.49^c^ 3.7	1.99±5.98^d^ 0.00	7.19±9.16^c^ 2.82	0.99±2.09^d^ 0.00	4.13±7.19 0.94	<0.001 *

*Kruskal Wallis γ Anova; a, b: lowercase equal in the same line indicates statistically similar data by Tukey test; c, d: lowercase equal in the same line indicates statistically similar data by Mann-Whitney test; Bonferroni correction PD – periodontal pocket depth; CAL – clinical attachment level

CP - control group with periodontitis

CNP - control group without periodontitis

BP - bariatric group with periodontitis

BNP - bariatric group without periodontitis

The first set of results derived from the microbial analysis of oral samples. In periodontitis subjects, *P. gingivalis* and *T. denticola* were significantly higher in the saliva samples taken from the bariatric group compared with the control group, suggesting that bariatric surgery was accompanied by higher salivary frequency of these two species in this specific periodontal status. The BP group also showed higher frequency of a third periodontopathic species, *T. forsythia*, but in the tongue dorsum instead (Table 2).

**Table 2:** Frequencies of bacteria, based on percentage (%) and number (n) of positive individuals, in saliva and/or tongue samples from control groups or bariatric groups according to periodontal status

		CP	CNP	BP	BNP	Total n=154	p-value
		% (n)	% (n)	% (n)	% (n)	% (n)	
Saliva	Hp	42.86 (15)	50.00 (20)	35.00 (14)	35.90 (14)	40.91 (63)	0.493^*^
	Pg	5.71 (2)^a^	7.50 (3)	30.00 (12)^a^	15.38 (6)	14.94 (23)	0.011^*^
	Aa	2.86 (1)	5.00 (2)	2.50 (1)	0.00	2.60 (4)	0.742^γ^
	Pm	77.14 (27)	65.00 (26)	67.50 (27)	56.41 (22)	66.23 (102)	0.308^*^
	Td	20.00 (7)	7.50 (3)^a^	35.00 (14)^a^	25.64 (10)	22.08 (34)	0.027^*^
	Tf	48.57 (17)	47.50 (19)	60.00 (24)	53.85 (21)	52.60 (81)	0.671^*^
	Cr	91.43 (32)^a^	57.50 (23)^a.b^	90.00 (36)^b^	76.92 (30)	78.57 (121)	0.001^*^
	Red Complex	2.86 (1)	2.50 (1)	17.50 (7)	10.26 (4)	8.44 (13)	0.074^γ^
Tongue	Hp	40.00 (14)	42.50 (17)	30.00 (12)	23.08 (9)	33.77 (52)	0.239^*^
	Pg	0.00	2.50 (1)	10.00 (4)	10.26 (4)	5.84 (9)	0.120^γ^
	Aa	0.00	2.50 (1)	5.00 (2)	0.00	1.95 (3)	0.619^γ^
	Pm	82.86 (29)^d^	57.50 (23)	72.50 (29)	51.28 (20)^d^	65.58 (101)	0.017^γ^
	Td	11.43 (4)	5.00 (2)	17.50 (7)	10.26 (4)	11.04 (17)	0.354^γ^
	Tf	45.71(16)	32.50 (13)^a^	67.5 (27)^a^	40.00 (16)	46.75 (72)	0.013^*^
	Cr	88.57 (31)^a^	50.00 (20)^a.b^	72.50 (29)	82.05 (32)^b^	72.73 (112)	0.001^*^
	Red Complex	0.00	2.50 (1)	5.00 (2)	2.56 (1)	2.60 (4)	0.903^γ^
Saliva and Tongue	Hp	42.86 (15)	55.00 (22)	35.00 (14)	41.03 (16)	43.51 (67)	0.330^*^
	Pg	5.71 (2)^a^	7.50 (3)^b^	32.50 (13)^a.b^	20.51 (8)	16.88 (26)	0.005^*^
	Aa	2.86 (1)	5.00 (2)	5.00 (2)	0.00	3.25 (5)	0.654^γ^
	Pm	91.43 (32)^a^	77.50 (31)	87.50 (35)	66.67 (26)^a^	80.52 (124)	0.031^*^
	Td	20.00 (7)	10.00 (4)a	37.50 (15)a	25.64 (10)	23.38 (36)	0.032^*^
	Tf	60.00 (21)	52.50 (21)	75.00 (30)	58.97 (23)	61.69 (95)	0.204^*^
	Cr	97.14 (34)^a^	62.50 (25)^a.b.c^	95.00 (38)^b^	92.31 (36)^c^	86.36 (133)	<0.001^γ^
	Red Complex	2.86 (1)	2.50 (1)	17.50 (7)d	12.82 (5)	8.44 (14)	0.010^γ^

* Test c2; γ Fisher's test, ** Red complex; a, b, c: lowercase equal in the same line indicates statistically different data by Pearson's c2 test; d: lowercase equal in the same line indicates statistically different data by Fisher's test; Bonferroni correction

*Hp = H. pylori - Pm = P. micra - Cr = C. rectus - Pg = P. gingivalis - Td = T. denticola - Aa =A. actinomycetemcomitans - Tf = T. forsythia*

CP - control group with periodontitis

CNP - control group without periodontitis

BP - bariatric group with periodontitis

BNP bariatric group without periodontitis

Interestingly, even in the non-periodontitis groups, the BNP group exhibited a higher frequency of *C. rectus* than that which usually precedes the colonization of the mouth by the red complex species. This bacterium was again observed in higher frequency in the saliva and tongue samples taken from the bariatric group. Individuals without periodontitis also showed the only significantly reduced frequency when bariatric and control groups were compared: *P. micra* appeared in lower frequency in the saliva taken from bariatric subjects.

Furthermore, the analysis of the oral representation brought a new perspective to the saliva/tongue isolated results, since each individual patient was considered through positive bacterial presence in at least one of the two sampled sites. Thus, the oral representation analysis corroborated the data separately found in the saliva and tongue samples for *P. gingivalis, T. denticola, T. forsythia, C. rectus,* and *P. micra*. The bariatric periodontitis group showed a higher frequency of *P. gingivalis, T. denticola, T. forsythia,* and *C. rectus.* The red complex analysis confirmed this overall tendency of a higher frequency of the target species in the mouths of postoperative bariatric subjects.

A high oral frequency of *H. pylori* was observed in the periodontitis and non-periodontitis groups, both in the saliva and dorsum of the tongue, ranging from 40.0% to 50.0% among the controls. Comparisons between the bariatric and control group with the same periodontal status did not reveal statistically significant differences. The analysis based on the oral representation criterion confirmed this lack of difference.

Along with the oral analysis, a second set of results that was derived from the stomach biopsies processed in the laboratory is further presented. The first surprising finding was the generally high frequency of well-known periodontal pathogens in both the bariatric (up to 83.3%) and control (up to 91.7%) groups. In the stomach, despite the health/disease status of the periodontium, nearly all periodontal pathogens showed lower significant frequencies in the bariatric group. Thus, in the long term (at least 24 months after surgery), bariatric surgery was accompanied by a reduced frequency of periodontal pathogens, as well as *H. pylori* in the stomach. Hence, bariatric surgery showed an inverse effect in oral and stomach microbial profiles.

Rather unexpectedly, the accuracy analysis (Table 3) demonstrated that the frequency observed in the stomach was similar to that observed in the mouth. However, the presence of *H. pylori* was higher in the stomach than in the mouth, as it was expected.

**Table 3:** Accuracy and bacteria frequencies, based on percentage (%) and number (n) of positive individuals, in stomach biopsies samples from control groups or bariatric groups according to periodontal status

		CP	CNP	BP	BNP	Total n=154	p-value	p- value Accuracy**
		% (n)	% (n)	% (n)	% (n)	% (n)		
Stomach	Hp	83.33 (10)^a^	50.00 (6)	75.00 (9)^b^	15.38 (2)^a.b^	55.10 (27)	0.003^*^	<0.001^*^
	Pg	58.33 (7)	33.33 (4)	75.00 (9)^c^	7.69 (1)^c^	34.69 (17)	0.049^γ^	0.467^γ^
	Aa	58.33 (7)^c^	25.00 (3)	41.67 (5)	0.00c	30.61 (15)	0.006^γ^	0.306^γ^
	Pm	83.33 (10)	91.67 (11)^c^	83.33 (10)	38.46 (5)^c^	73.47 (36)	0.015^γ^	0.650^γ^
	Td	83.33 (10)^a^	83.33 (10)^b^	50.00 (6)	15.38 (2)^a.b^	57.14 (28)	0.001^*^	0.325^γ^
	Tf	66.67 (8)^a^	66.67 (8)^b^	50.00 (6)	7.69 (1)^a.b^	46.94 (23)	0.008^*^	0.070^γ^
	Cr	75.00 (9)	83.33 (10)^a^	83.33 (10)^b^	30.77 (4)^a.b^	67.35 (33)	0.018^γ^	0.073^γ^
	Red Complex	58.33 (7)^c^	33.33 (4)	41.67 (5)	0.00c	32.65 (16)	0.008^γ^	0.402^γ^

* Test c2; γ Fisher's test, ** Accuracy test with oral representation; a, b: lowercase equal in the same line indicates statistically different data by Pearson's c2 test; c: lowercase equal in the same line indicates statistically different data by Fisher's test; Bonferroni correction

*Hp = H. pylori - Pm = P. micra - Cr = C. rectus - Pg = P. gingivalis - Td = T. denticola - Aa =A. actinomycetemcomitans - Tf = T. forsythia*

CP - control group with periodontitis

CNP - control group without periodontitis

BP - bariatric group with periodontitis

BNP bariatric group without periodontitis

Table 4 shows the influence of *H. pylori* on the detection of the species periodontally sought. Some associations between *H. pylori* and oral species were found; for example, *P. gingivalis, T. denticola, T. forsythia,* and *C. rectus* were involved in these associations. Interestingly, these oral species were also related to statistically significant results observed in the previously conducted analysis. Among the subjects colonized by *H. pylori*, differences of combined *H. pylori* - *P. gingivalis* (Hp-Pg) frequency in saliva were found among the groups (p=0.033). This association showed a tendency for increased bacterial occurrence in bariatric subjects with periodontitis (BP group; p=0.021). In periodontitis subjects, the association between *H. pylori* and *T. forsythia* (Hp-Tf) was higher in the tongue samples, especially in the bariatric group (BP group; p=0.006). The association between *H. pylori* and *C. rectus* (Hp-Cr) was statistically higher in the bariatric groups with periodontitis (BP) than in the non-periodontitis bariatric (BNP) groups (p=0.006). In the previous analysis, *C. rectus* did not show any statistically significant results among individuals with periodontitis. However, *C. rectus* appeared again as a key pathogen in the non-periodontitis individuals. The association between *H. pylori* and *C. rectus* (Hp-Cr) was statistically higher in the bariatric group (BNP; p<0.001). Considering the oral representation, the data previously showed was confirmed. The red complex species still showed a significantly higher frequency in the bariatric group. Significant differences in the simultaneous frequency of *H. pylori* and periodontal pathogens in the stomach were not observed (data not shown).

**Table 4:** Simultaneous frequency, based on percentage (%) and number (n) of positive individuals, of H. pylori and periodontal pathogens in saliva and/or tongue samples from control groups or bariatric groups according to periodontal diagnosis

		CP	CNP	BP	BNP	Total	p-value
		% (n)	% (n)	% (n)	% (n)	% (n)	
Saliva		n=15	n=20	n=14	n=14	n=63	
	Pg	6.67 (1)	0.00 (0)	28.57 (4)	14.29 (2)	11.11 (7)	0.033^γ^
	Aa	6.67 (1)	10.00 (2)	7.14 (1)	0.00 (0)	6.35 (4)	0.901^*^
	Pm	86.67 (13)	55.00 (11)	71.43 (10)	57.14 (8)	66.67 (42)	0.195^γ^
	Td	20.00 (3)	5.00 (1)	14.29 (2)	28.57 (4)	15.87 (10)	0.315^γ^
	Tf	60.00 (9)	30.00 (6)	42.86 (6)	57.14 (8)	46.03 (29)	0.261^*^
	Cr	80.00 (12)	50.00 (10)	85.71 (12)	71.43 (10)	69.84 (44)	0.133^γ^
	Red Complex	0.00 (0)	0.00 (0)	0.00 (0)	14.29 (2)	3.17 (2)	0.093^γ^
Tongue		n=14	n=17	n=12	n=9	n=52	
	Pg	0.00 (0)	0.00 (0)	16.67 (2)	0.00 (0)	3.85 (2)	0.077^γ^
	Aa	0.00 (0)	5.88 (1)	16.67 (2)	0.00 (0)	5.77 (3)	0.337^γ^
	Pm	78.57 (11)	64.71 (11)	83.33 (10)	44.44 (4)	69.23 (36)	0.231^γ^
	Td	14.29 (2)	5.88 (1)	8.33 (1)	0.00 (0)	7.69 (4)	0.821^γ^
	Tf	50.00 (7)	23.53 (4)^a^	75.00 (9)^a^	33.33 (3)	44.23 (23)	0.043^γ^
	Cr	78.57 (11)^a^	29.41 (5)^a.d^	75.00 (9)	100.00 (9)^d^	65.38 (34)	0.001^γ^
	Red Complex	0.00 (0)	0.00 (0)	0.00 (0)	0.00 (0)	0.00 (0)	-
Saliva and Tongue		n=15	n=22	n=14	n=16	n=67	
	Pg	6.67 (1)	0.00 (0)^a^	35.71 (5)^a^	25.00 (4)	14.93 (10)	0.005^γ^
	Aa	6.67	9.09 (2)	14.29 (2)	0.00 (0)	7.46 (5)	0.458^γ^
	Pm	93.33 (14)	77.27 (17)	92.86 (13)	75.00 (12)	83.58 (56)	0.365^γ^
	Td	20.00 (3)	9.09 (2)	21.43 (3)	25.00 (4)	17.91 (12)	0.572^γ^
	Tf	73.33 (11)	40.91 (9)	71.43 (10)	56.25 (9)	58.21 (39)	0.161^*^
	Cr	93.33 (14)^a^	40.91 (9)^a.b.c^	92.86 (13)^b^	100.00 (16)^c^	77.61 (22)	<0.001^γ^
	Red Complex	0.00 (0)^a^	0.00 (0)^b^	0.00 (0)^c^	18.75 (3)^a.b.c^	4.48 (3)	0.029^γ^

* Test c2; γ Fisher's test, ** Red complex; a, b, c: lowercase equal in the same line indicates statistically different data by Pearson's c2 test; d: lowercase equal in the same line indicates statistically different data by Fisher's test; Bonferroni correction

*Hp = H. pylori - Pm = P. micra - Cr = C. rectus - Pg = P. gingivalis - Td = T. denticola - Aa =A. actinomycetemcomitans - Tf = T. forsythia*

CP - control group with periodontitis

CNP - control group without periodontitis

BP - bariatric group with periodontitis

BNP bariatric group without periodontitis

When evaluating the influence of age and tobacco use in the groups, there were no statistically significant differences, either in relation to bariatric surgery or to the presence or absence of periodontitis.

## DISCUSSION

Evidence suggests a two-way relationship between periodontal disease and obesity^20^ ^,^ ^22^. In addition, a previous study that analyzed 345 subjects undergoing bariatric surgery showed a high prevalence of periodontitis, ranging from 70.69% to 91.66% of individuals before and after bariatric surgery^23^. Up to now, few studies had evaluated the relationship between periodontal pathogens and obesity; therefore, the present findings of the study are particularly revealing. A relevant and high frequency of periodontal pathogens and *H. pylori* in both oral and stomach environments was demonstrated. The high frequency of *H. pylori* observed was expected^8^. However, the high occurrence of oral species in the stomach was rather intriguing.

It has been suggested that obesity in humans can increase the risk of periodontitis^21^ and also that oral biofilms play a fundamental role in oral-systemic medicine^27^. Haffajee and Socransky^11^ (2009) found a significantly higher frequency of *T. forsythia* in obese individuals with gingivitis. Moreover, Goodson, et al.^10^ (2009) found changes in the salivary bacterial composition in overweight women while Matsushita, et al.^17^ (2015) reported that the red complex bacterial species, i.e., *P. gingivalis, T. denticola,* and *T. forsythia*, are associated with obesity. In our study, the presence of *T. forsythia* (up to 72.2%), *P. gingivalis* (up to 30.0%), *T. denticola* (up to 35.0%), *C. rectus* (up to 91.4%), and *P. micra* (up to 82.9%) were verified in high frequency in the mouth of the bariatric and control groups. The only periodontal species that was always observed in low frequency (up to 5.0%) was *A. actinomycetemcomitans*. Although these findings are not new, the present study is able to corroborate the aforementioned theory in which obesity modifies the immune host response, which, therefore, influences human microbiota. According to Nagpal, et al.^20^ (2015), the penetration of periodontal pathogens or their products in lamina propria may lead to endotoxemia and to a state of systemic chronic inflammation. This state may further affect the expression and functioning of important immunoinflammatory molecules, thereby contributing to altered lipid and glucose metabolisms. In our study, the bariatric group showed an increased frequency of the red complex species. These findings, as well as those of Haffajee and Socransky^11^ (2009), suggest the need for longitudinal monitoring of obese patients, since these bacteria play a key role in the development and progression of periodontal disease. It is known that weight reduction may benefit overweight and obese people in particular, mainly due to the associated reduction in their inflammatory responses^4^, but also because it can positively impact their periodontium^20^ ^,^ ^25^. Furthermore, the control of comorbidities, such as diabetes, could also lead to a reduction of periodontal pathogens. In the present study, most of the target species appeared in greater numbers in the bariatric group, and they were even more evident among individuals with periodontitis. However, it is important to emphasize that the intense and progressive changes that occur during the first few years after the surgery could delay the establishment of the oral microbial profile. Unfortunately, despite the systemic benefits of bariatric surgery, oral disorders, such as the loss of periodontal tonus, bleeding, hypersensitivity and xerostomia, have been related to this form of treatment for obesity.

One surprising observation of this study was the high frequency of periodontal pathogens present in the biopsies of stomach tissue, with some bacterial frequency reaching over 90%. Even species such as *A. actinomycetemcomitans* (58.3%), which is sometimes only found in low numbers in the mouth, were commonly detected in the stomach. In addition, although many *H. pylori* infections are treated before surgery using systemic antibiotics, in our study this microorganism was frequently found in stomachs and in the mouths of the bariatric and control groups. Overall, longitudinal studies are required to further elucidate oral and stomach bacterial profiles in response to bariatric surgery. In addition, future longitudinal studies could investigate periodontitis in obese patients before and after bariatric surgery. Currently, long-term studies on the topic are scarce. In an unselected population based on periodontal status, Jaiswal, et al.^12^ (2015) failed to report pocket depth and clinical attachment level improvements in a six-month monitoring period after bariatric surgery. Similarly, Sales-Peres, et al.^24^ (2015) observed worsened measurements of pocket depth and clinical attachment levels six months after bariatric surgery, alongside an increased amount of *P. gingivalis.*

Although the primary site of *H. pylori* colonization is the stomach, the mouth also harbors this pathogen, even if temporarily, mainly in individuals with chronic gingivitis or periodontitis^2^ ^,^ ^9^ ^,^ ^28^. Thus, the oral cavity could represent an extragastric reservoir of *H. pylori* ^9^ ^,^ ^28^. Interestingly, the professional control of dental biofilm is associated with lower gastric reinfection of *H. pylori*. In a study involving 110 individuals, only 19.6% of patients who received oral biofilm control were reinfected by *H. pylori* compared with 84.3% of patients without professional biofilm control^13^. In our study, there was a high frequency of *H. pylori* in the subjects’ stomachs (from 15.3 to 83.3%), saliva, and tongues (from 30.0 to 50.0%). In the control group, individuals who harbored this bacterium in the stomach were more likely to carry it in their mouths as well. These patients were probably not successfully treated for *H. pylori* infection and they had higher BMIs than individuals from the bariatric group. Our findings confirm previous reports of a higher prevalence *of H. pylori* in obese patients than in non-obese patients^1^. Erim, et al.^8^ (2008) also found that bariatric surgically treated patients were 1.7 times more likely (95% CI, 1.3 - 2.2) to demonstrate *H. pylori* infection. In general terms, our study showed a simultaneous frequency of *H. pylori* and periodontal pathogens in both the periodontitis groups and in the bariatric groups.

Tests of accuracy between the presence of periodontal pathogens in the stomach and mouth were not statistically significant. The stomach is the primary site of *H. pylori,* which can help explain its statistically higher frequency in the stomach than in the mouth. Similarly, periodontal pathogens demonstrated higher frequency in their primary oral sites when compared with the stomach.

The relationship between microorganisms and obesity is not yet well understood. This complex and intriguing relationship reveals several possibilities in a wide field of research. Therefore, future research to examine how oral bacteria can influence obesity is strongly recommended. Finally, the possible mouth-stomach route highlighted in this study draws attention to other ways for periodontal pathogens to migrate from oral to systemic sites. It is important to consider that this route is not necessarily dependent on the diseased epithelium found in the walls of inflamed gingival/periodontal pockets. Once more, the maintenance of local health and control of oral microbiota appear to impact a person’s systemic health. Considering the possible identification of DNA from non-viable bacteria, the authors also suggest the use of a sample analysis using different microbial techniques, especially quantitative ones. However, under the correct conditions, a small bacterial fragment or even bacterial products could initiate and/or sustain an inflammatory response.

Some limitations of our study, such as the use of a convenience sample, should also be mentioned. The most obvious criticism about convenience sampling is that the sample is not representative of the entire population, which limits data generalization. In addition, because of their overall compromised systemic condition, the exclusion of morbidly obese participants could have impacted the results drawn from the present study, although we cannot presume to know what type of microbial interactions among oral bacteria and *H. pylori* would be expected in this group. Moreover, a greater number of stomach biopsies should be microbiologically analyzed by using quantitative techniques to determine any infective patterns. Some of the limitations observed in the study could be corrected in future research by randomly selecting obese patients to more accurately represent the entire population and by including other degrees of obesity and quantifying bacterial levels.

Our study suggests that the stomach, although a different environment from the oral cavity, can harbor specific oral bacteria. For now, we do not know whether oral bacteria in the stomach are able to migrate to other parts of the body and if periodontal pathogens can contribute to stomach disease in obese or bariatric surgically treated individuals. The results of this study showed that obese individuals had higher levels of periodontal pathogens and *H. pylori* in both their mouths and stomachs. It is clear that bariatric surgery has influenced bacterial frequency in these environments, but the changes that occur after the surgery seem to trigger distinctive effects in the mouth and in the stomach.

## CONCLUSIONS

Bariatric surgery showed an inverse microbial effect on oral and stomach environments and was accompanied by higher oral and lower stomach bacterial frequencies.
